# A Cardioprotective perfusion protocol limits myocardial functional decline during ex situ heart perfusion

**DOI:** 10.1016/j.jhlto.2023.100042

**Published:** 2023-12-20

**Authors:** Mats T. Vervoorn, Elisa M. Ballan, Sjoerd van Tuijl, Saskia C.A. de Jager, Selma E. Kaffka genaamd Dengler, Joost P.G. Sluijter, Pieter A. Doevendans, Niels P. van der Kaaij

**Affiliations:** a University Medical Center Utrecht, Department of Cardiothoracic Surgery, Division of Heart & Lungs, Utrecht, the Netherlands; b University Medical Center Utrecht, Department of Cardiology, Laboratory of Experimental Cardiology, Division Heart & Lungs, Utrecht, the Netherlands; c Netherlands Heart Institute, Utrecht, the Netherlands; d LifeTec Group B.V., Eindhoven, the Netherlands; e Regenerative Medicine Utrecht, Circulatory Health Research Center, University Utrecht, Utrecht, the Netherlands; f University Medical Center Utrecht, Department of Cardiology, Division Heart & Lungs, Utrecht, the Netherlands

**Keywords:** ex situ heart perfusion, heart transplantation, heart failure, cardiac surgery, organ preservation, machine perfusion

## Abstract

**Background:**

Ex situ heart perfusion is associated with a significant decline in graft quality related to oxidative stress, inflammation, endothelial dysfunction, and metabolic perturbations. We assessed the effects of a more optimized, cardioprotective normothermic perfusion approach compared to a conventional perfusion protocol in a slaughterhouse model using porcine hearts.

**Methods:**

A total of 12 hearts were harvested and subjected to 4 hours of normothermic perfusion. The optimized protocol consisted of an adenosine-lidocaine cardioplegic solution, subnormothermic initial reperfusion and controlled rewarming, hemofiltration and supplementation of methylprednisolone and pyruvate. This was compared to a conventional protocol consisting of St. Thomas II cardioplegic solution, normothermic initial reperfusion without hemofiltration or methylprednisolone, and a mixture of glucose and insulin for metabolic support.

**Results:**

Myocardial function was superior in the optimized group, while significant functional decline was absent. Hearts subjected to the conventional protocol demonstrated a significant reduction in function over time.

**Conclusions:**

We have developed a further optimized, cardioprotective normothermic ex situ heart perfusion approach and demonstrated significantly improved myocardial function and attenuated functional decline during 4 hours of normothermic perfusion, indicating improved preservation.

## Background

Normothermic ex situ heart perfusion (ESHP) has emerged as a promising technique for resuscitation and quality assessment of donor hearts in clinical heart transplantation.^1^, ^2^ However, a common observation during ESHP is the decline in myocardial function and graft quality, which is linked to metabolic perturbations, induced inflammatory response, increased oxidative stress, and ischemia-reperfusion injury.^3^, ^4^, ^5^, ^6^, ^7^ These events contribute to a vicious cycle of energy deficiency, endothelial cell activation, and edema formation that ultimately results in graft failure.^3^, ^4^, ^5^, ^6^, ^7^ It is imperative to break this pathophysiological cascade to broaden the potential applications of ESHP, from extended graft preservation for clinical transplantation to application of techniques (i.e., gene therapy^8^) for biological modification.^1^

In several studies, interventions that target specific pathophysiologic pathways during ESHP have been applied to improve myocardial preservation and cardioprotection. These include reperfusion strategies to ameliorate ischemia-reperfusion injury,^3^, ^9^, ^10^ improve perfusate composition,^11^, ^12^ reduce the associated inflammatory response and edema formation,^13^ optimized loading conditions,^7^, ^14^ and metabolic substrate selection.^5^, ^14^ Some of them have shown favorable effects on preserving myocardial function, but the potential synergistic effect of these interventions together has yet to be assessed.

**Figure 1:**
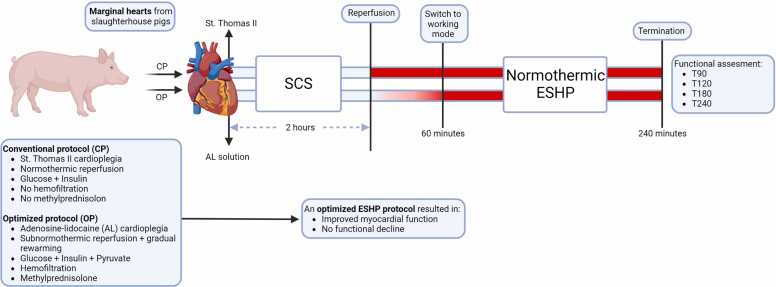
Experimental design and most important results. CP, conventional protocol; OP, optimized protocol; SCS, static cold storage; ESHP, ex situ heart perfusion; T, timepoint in minutes. The figure was constructed with Biorender.

This study aims to evaluate the effectiveness of a further optimized protocol (OP) with cardioprotective properties for normothermic ESHP, combining an adenosine-lidocaine cardioplegic solution, subnormothermic initial reperfusion and controlled rewarming, hemofiltration and supplementation of methylprednisolone and pyruvate in a slaughterhouse model using marginal porcine hearts.^15^ The efficacy of the OP will be compared to a control group of hearts subjected to a conventional ESHP protocol (CP), consisting of a commonly-used cardioplegic solution (St. Thomas II), normothermic initial reperfusion, without hemofiltration or methylprednisolone added, and a mixture of glucose and insulin for metabolic support (Figure 1).

## Material and methods

### Animals

A total of 12 hearts were harvested from Dutch Landrace Hybrid pigs slaughtered for human consumption. The protocols were consistent with regulation number 1069/2009 of the European Parliament and Council regarding slaughterhouse animal material for diagnosis and research, which were authorized by the relevant legal animal welfare authorities (Food and Consumer Product Safety Authority).

### Heart procurement and cold preservation

Before heart procurement, the pigs were electrically stunned, hanged, and exsanguinated, as described previously.^15^ After parasternal incision, the heart and lungs were harvested en-bloc, and the aorta was cannulated before administering a cooled (4°C) cardioplegic solution at a pressure of 80 to 100 mm Hg. The cardioplegic solution administered differed between the CP and OP groups according to the applied protocol (Table 1). Autologous blood was collected during exsanguination using a collection bag primed with 5000 IU of heparin. The hearts were stored on ice for 2 hours submerged in cold cardioplegic solution during transport, before undergoing reperfusion.

**Table 1:** Overview of the Different Perfusion Protocols Used

Conventional protocol	Optimized protocol
*Phase 1: cardioplegic solution*	
St. Thomas II	Adenosine-lidocaine solution
*Phase 2: initial reperfusion*	
Normothermic reperfusion (38°C)	Subnormothermic reperfusion (28°C) and gradual rewarming
*Phase 3: normothermic perfusion*	
No hemofiltration	Hemofiltration (1000 ml/hour)
No methylprednisolon	Methylprednisolon (1000 mg)
Glucose + insulin	Glucose + insulin + pyruvate (12.5 mmol/liter/hour)

### Ex situ heart perfusion

ESHP was conducted using the PhysioHeart platform (LifeTec Group, Eindhoven), a system that enables both loaded and unloaded perfusion and functional cardiac assessment.^16^, ^17^ Prior to reperfusion, the system was primed with 1.5 liter of Krebs-Henseleit buffer. Previously collected autologous heparinized whole blood was added to reach a total perfusion volume of 4.5 liter, with hemoglobin levels ranging between 3.0 and 4.5 mmol/liter. A gas mixture of oxygen (FiO_2_ 0.25) and carbon dioxide was used to maintain a pO_2_ of 100 to 200 mm Hg and pCO_2_ of 35 to 45 mm Hg. The biochemical composition of the perfusate was monitored using a VetScan iSTAT 1 and CG8+ cartridges (Abbott Laboratories. Chicago, IL) and maintained in a normo-physiologic range (pH: 7.35-7.34; HCO_3–_: 20-25 mmol/liter; Na+: 135-145; K+: 3.0-5.0; Ca^2+^: 1.25 mmol/liter). After initial reperfusion, the first 500 ml reperfusate was collected and the hearts were subjected to unloaded perfusion for 60 minutes. Atrio-ventricular sequential pacing was commenced via the right atrium and right ventricle at a rate of 100 to 115 beats-per-minute, depending on the amount of interference with the heart’s intrinsic heart rate and the most optimal hemodynamic performance. In case of ventricular arrhythmias after reperfusion, hearts were defibrillated with 10 to 50 J, lidocaine (10 mg), and magnesium sulfate (500 mg) were administered. After 60 minutes of unloaded perfusion, the hearts were subjected to an additional 3 hours of loaded perfusion. Loading conditions were standardized: left atrial pressure (LAP) was maintained between 10 and 15 mm Hg, and afterload was adjusted according to a predefined ratio between mean aortic pressure (MAP) and cardiac output (CO) (i.e., every incremental liter of CO is accompanied by an increase of 10 mm Hg MAP; i.e., a CO of 3.0 liter/minutes corresponds to an MAP of 60 mm Hg) to attain physiologic values. This was based on prior research with PhysioHeart.^18^

### Experimental groups

#### Conventional protocol (CP)

After harvesting, the hearts were submerged in cold saline and 2 liter of St. Thomas II solution was infused. Following static cold storage, perfusion at 38°C was commenced with a mixture of 1.5 liter KHB and 3 liter heparinized autologous whole blood for a total ESHP duration of 4 hours (Figure 1).

#### Optimized protocol (OP)

After procurement, the hearts were submerged in cold saline and 2 liter of a normokalemic adenosine-lidocaine (200/500 mcg/liter) cardioplegic solution was infused, containing a mixture of glucose (10 mmol/liter), insulin (1 IU/liter), pyruvate (1 mmol/liter), and rendered hypocalcemic (0.6 mmol/liter). Following static cold storage, reperfusion at 28°C at a perfusion pressure of 50 mm Hg was commenced with a mixture of 1.5 liter KHB, supplemented with methylprednisolone (1000 mg; Solu-Medrol, Pfizer), and 3 liter heparinized autologous whole blood for a total ESHP duration of 4 hours. Pyruvate (5 mmol/liter) was added to the perfusate solution before reperfusion. At the time of reperfusion, hemofiltration was initiated at a rate of 1000 ml/hour, and the ultrafiltrate was replaced in a 1:1 ratio with KHB supplemented with pyruvate (12.5 mmol/liter of total circulating perfusate). After the start of reperfusion, the perfusate was rewarmed to 38°C over 45 minutes, with incremental steps of 0.8°C every 3 minutes. The perfusion pressure was also incrementally increased by 10 mm Hg every 15 minutes during controlled rewarming, up to a perfusion pressure of 80 mm Hg. ESHP was continued for a total duration of 4 hours (Figure 1).

### Functional assessment

Functional assessment was conducted in working mode at different timepoints (90, 120, 180, and 240 minutes of perfusion). We opted for our first functional measurement at 90 minutes as this allowed the hearts to adequately recover from cold preservation and subsequent myocardial stunning. Data were recorded for 10 seconds at a sampling rate of 1000 Hz. The recorded data included time in seconds, CO, coronary flow, left ventricular pressure (LVP), LAP, and MAP. To obtain LVP, a pressure wire (PressureWire, Radi Medical Systems, Uppsala, Sweden) was introduced transapically into the left ventricle, and dP/dT values were calculated based on LVP and time. CO and coronary flow were adjusted for heart weight to account for the influence of heart size, resulting in cardiac index (CI) and coronary flow index (CFI). LV stroke work index (LVSWI) was calculated using CO-derived stroke volume and LV developed pressure, divided by individual heart weight. Dobutamine was infused at incremental doses of 1.2 ml/hour, starting at 2.4 ml/hour and increased to a maximum of 6 ml/hour when CO decreased below 3.5 liter/min and LAP increased to >15 mm Hg. The dobutamine requirement per heart was recorded and included as an indirect marker of myocardial function, as a higher requirement reflects a decreased function.

### Statistical analysis

Data analysis was performed using GraphPad Prism (version 9.3.0 for Windows. GraphPad Software, San Diego, CA). Between group comparison was conducted using Mann-Whitney U test, within group analysis for differences over time (T90 vs T240) was conducted using a Wilcoxon matched-pairs signed rank test. Data are presented as median and interquartile range. *p*-values <0.05 were deemed statistically significant.

## Results

### Harvesting time, heart weight, weight gain, and defibrillation requirements

No significant difference was observed in harvesting time (190 vs 195 seconds) and heart weight (388 vs 393 g) between the OP and CP groups. Hearts in the OP group numerically gained less weight over the course of normothermic perfusion compared to the CP group (9.6% vs 17.1%), although this was not statistically significant. The defibrillation requirement after reperfusion was, however, significantly higher in the CP group (0 vs 3, *p* = 0.002; Table 2).

**Table 2:** Baseline Characteristics

Variable	OP	CP	*p*-value
Number of hearts	6	6	N/A
Harvesting time (second), median (IQR)	195 ± 10	190 ± 25	0.696
Harvesting weight (gram), median (IQR)	388 (362-453)	393 (349-473)	0.775
Weight gain (gram), median (IQR)	34 (12-62)	70 (30-124)	0.260
Weight gain (% of baseline), median (IQR)	9.6 (2.6-15.9)	17.1 (5.2-31.7)	0.394
Defibrillation at reperfusion (#), median (IQR)	0 (0-1)	3 (2-4.3)	0.002^a^

Abbreviations: CP, conventional protocol; IQR, interquartile range; N/A, not applicable; OP, optimized protocol.

a Indicates statistical significance.

### Myocardial function is superior with the further optimized, cardioprotective protocol, while functional decline is minimal

**Figure 2:**
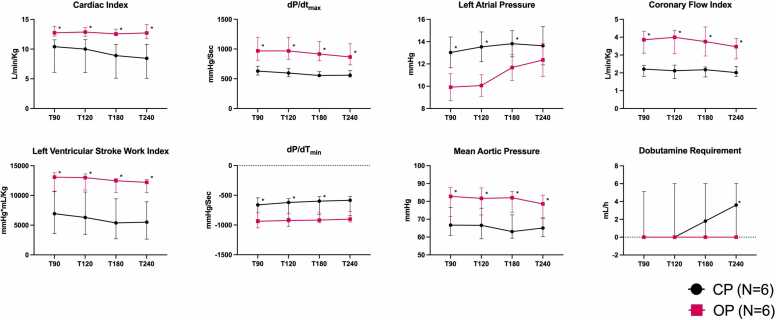
Myocardial function over the course of perfusion. CP, conventional protocol; h, hour; Kg, kilogram; L, liter; Min, minutes; ml, milliliter; OP, optimized protocol; T, timepoint in minutes.

Over the course of perfusion, the OP group displayed significantly higher values of CI, LVSWI, dP/dT-values and CFI, as compared to the CP group (Figure 2). This was achieved at significantly lower LAP and significantly higher MAP in the OP group (Table 3), indicative of improved function. Dobutamine requirement was also significantly higher in the CP group compared to the OP group.

**Table 3:** Myocardial Function

Variable	Group	T90	T120	T180	T240	*p*-value
CI (liter/min/kg), median (IQR)	OP	12.7 (12.2-13.8)	12.9 (12.2-13.6)	12.6 (12.3-13.4)	12.7 (11.7-14.2)	0.448
	CP	10.4 (6.1-11.6)	10.0 (6.1-11.6)	8.9 (5.1-10.8)	8.5 (5.1-10.8)	0.032^a^
	*p*-value	0.041^a^	0.041^a^	0.041^a^	0.026^a^	
LVSWI (mm Hg*ml/kg), median (IQR)	OP	13,086 (10,624-13,806)	12,999 (10,914-13,627)	12,469 (10,520-12,837)	12,210 (10,471-12,691)	0.564
	CP	6,933 (3,581-10,717)	6,292 (3,409-10,578)	5,376 (1,708-9,430)	5,504 (2,641-8,936)	0.063
	*p*-value	0.015^a^	0.0151^a^	0.026^a^	0.026^a^	
LAP (mm Hg), median (IQR)	OP	10.3 (8.8-10.9)	10.4 (9.22-10.7)	12.0 (10.3-12.6)	12.5 (11.3-13.4)	0.031^a^
	CP	12.8 (11.8-14.5)	13.9 (12.0-14.8)	14.4 (12.5-14.8)	14.2 (12.1-14.9)	0.250
	*p*-value	0.002^a^	0.002^a^	0.036^a^	0.195	
dP/dT_max_ (mm Hg/sec), median (IQR)	OP	970 (812-1,199)	968 (829-1,199)	917 (805-1,128)	866 (734-1,090)	0.156
	CP	629 (564-712)	597 (535-670)	555 (528-621)	561 (530-639)	0.156
	*p*-value	0.02^a^	0.004^a^	0.004^a^	0.004^a^	
dP/dT_min_ (mm Hg/sec), median (IQR)	OP	937 (794-1,047)	922 (801-1,024)	917 (797-961)	900 (771-947)^a^	0.313
	CP	661 (542-907)	620 (555-885)	598 (521-826)	584 (516-843)	0.031^a^
	*p*-value	0.041^a^	0.041^a^	0.041^a^	0.065	
CFI (liter/min/kg), median (IQR)	OP	3.9 (3.1-4.3)	4.0 (3.1-4.4)	3.8 (2.9-4.6)	3.5 (2.8-3.9)	0.063
	CP	2.2 (1.8-2.4)	2.1 (1.7-2.2)	2.2 (1.8-2.3)	2.0 (1.8-2.4)	0.563
	*p*-value	0.002^a^	0.002^a^	0.002^a^	0.009^a^	
MAP (mm Hg), median (IQR)	OP	82.8 (71.5-87.8)	81.7 (72.4-87.6)	82.0 (73.9-85.6)	79 (71-83)	0.313
	CP	66.7 (60.9-76.8)	66.6 (59.0-76.2)	63.1 (59.4-72.4)	65.1 (60.2-70.4)	0.375
	*p*-value	0.026^a^	0.063	0.015^a^	0.026^a^	
Dobutamine requirement (ml/hour), median (IQR)	OP	0.0 (0.0-0.0	0.0 (0.0-0.0	0.0 (0.0-0.0	0.0 (0.0-0.0	N/A
	CP	0.0 (0.0-5.1)	0.0 (0.0-6.0)	1.8 (0.0-6.0)	3.6 (0.0-6.0)	0.250
	*p*-value	0.18	0.17	0.09	0.04^a^	

Abbreviations: CFI, coronary flow index; CI, cardiac index; CP, conventional protocol; IQR, interquartile range; LAP, left atrial pressure; LVSWI, left ventricular stroke work index; MAP, mean aortic pressure; N/A, not applicable; OP: optimized protocol.

a Indicates statistical significance.

Furthermore, a declining trend in function between T90 and T240 was noted in the CP group as indicated by a significantly declining CI (*p* = 0.032), a declining LVSWI (*p* = 0.063) and a significantly declining dP/dT_min_ (*p* = 0.031; Table 3). This was not the case in the OP group (Figure 2). The only sign of functional decline noted in the OP group was a slow increase in LAP over the course of perfusion (10.3-12.5 mm Hg).

### Perfusate composition

**Figure 3:**
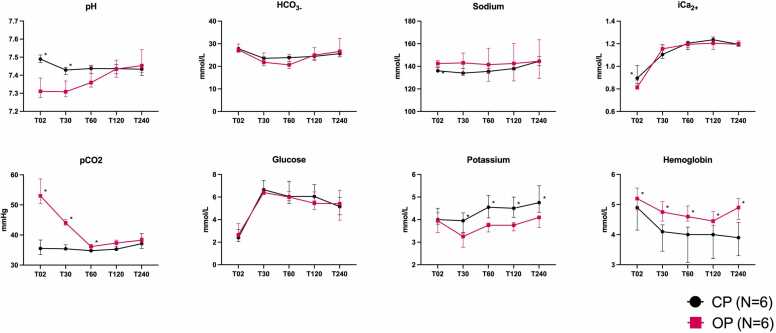
Bloodgas and biochemistry over the course of perfusion. pCO_2_, partial pressure of CO_2_.

At baseline, sodium was significantly higher in the OP group (136 vs 143 mmol/liter; *p* = 0.011) due to the added sodium pyruvate. During rewarming, pH and pCO_2_ values were significantly higher in the OP group. Over the course of perfusion, pO_2_, pCO_2_, pH, sodium, ionized calcium, and glucose levels did not differ between groups. However, potassium levels were significantly lower and hemoglobin levels significantly higher in the OP group at all timepoints (Table 4; Figure 3).

**Table 4:** Bloodgas and Biochemistry Values During Normothermic Perfusion

Variable	Group	T02	T30	T60	T120	T240
pH, median (IQR)	OP	7.31 (7.28-7.38)	7.31 (7.28-7.37)	7.36 (7.33-7.46)	7.43 (7.39-7.48)	7.45 (7.42-7.54)
	CP	7.49 (7.47-7.51	7.43 (7.40-7.44)	7.44 (7.41-7.45)	7.44 (7.41-7.46)	7.43 (7.40-7.45)
	*p*-value	0.002^a^	0.002^a^	0.13	0.94	0.24
pCO_2_ (mm Hg), median (IQR)	OP	53.0 (50.4-58.6)	44.1 (43.3-45.2)	36.2 (35.8-36.9)	37.4 (36.2-38.3)	38.3 (36.9-39.0)
	CP	35.6 (33.6-38.3)	35.4 (34.9-36.7)	34.8 (34.6-35.8)	35.3 (35.0-37.2)	37.1 (35.5-40.5)
	*p*-value	0.002^a^	0.002^a^	0.04^a^	0.09	0.78
pO_2_ (mm Hg), median (IQR)	OP	238 (229-262)	193 (177-210)	167 (153-185)	158 (141-173)	157 (137-173)
	CP	187 (170-225)	173 (159-195)	172 (154-194)	156 (138-173)	154 (129-172)
	*p*-value	0.02^a^	0.18	0.56	0.94	0.67
HCO_3–_ (mmol/liter), median (IQR)	OP	27.3 (26.3-29.9)	21.8 (20.3-25.9)	20.1 (19.0-25.4)	25.0 (22.5-28.3)	26.6 (25.1-32.2)
	CP	27.8 (26.5-28.0)	23.6 (22.5-24.2)	23.9 (22.2-24.5)	24.4 (22.9-25.3)	25.6 (24.2-26.0)
	*p*-value	0.86	0.48	0.17	0.85	0.20
Sodium (mmol/liter), median (IQR)	OP	143 (140-145)	143 (132-152)	143 (127-156)	143 (127-160)	145 (129-164)
	CP	136 (135-139)	134 (134-138)	136 (134-138)	138 (137-142)	145 (141-149)
	*p*-value	0.011^a^	0.511	0.851	0.999	0.999
Potassium (mmol/liter), median (IQR)	OP		4.0 (3.4-4.3)	3.8 (3.5-3.9)	3.8 (1.5-3.9)	4.1 (3.7-4.2)
	CP		3.3 (3.0-3.4)	4.6 (4.1-5.1)	4.5 (4.1-5.0)	4.8 (4.3-5.5)
	*p*-value	0.569	0.019^a^	0.019^a^	0.009^a^	0.015^a^
iCa^2+^ (mmol/liter), median (IQR)	OP	0.82 (0.80-0.88)	1.15 (1.15-1.19)	1.20 (1.17-1.21)	1.21 (1.15-1.23)	1.20 (1.19-1.23)
	CP	0.90 (0.85-1.01)	1.11 (1.07-1.19)	1.21 (1.15-1.22	1.24 (1.21-1.26)	1.20 (1.18-1.21)
	*p*-value	0.05^a^	0.22	0.86	0.10	0.86
Glucose (mmol/liter), median (IQR)	OP	2.7 (2.5-3.7)	6.4 (6.2-6.7)	6.0 (5.7-6.5)	5.5 (4.9-6.5)	5.4 (4.0-6.6)
	CP	2.4 (2.1-3.1)	6.7 (6.2-7.5)	6.1 (5.4-7.4)	6.1 (5.7-7.10)	5.2 (4.4-6.0
	*p*-value	0.24	0.48	0.97	0.14	0.61
Hemoglobin (mmol/liter), median (IQR)	OP	5.2 (4.8-5.6)	4.8 (4.5-5.1)	4.6 (4.5-5.0)	4.5 (4.3-4.8)	4.9 (4.5-5.2)
	CP	4.9 (4.2-4.9)	4.1 (3.5-4.3)	4.0 (3.1-4.3)	4.0 (3.2-4.4)	3.9 (3.3-4.4)
	*p*-value	0.05^a^	0.019^a^	0.017^a^	0.051	0.013^a^

Abbreviations: CP, conventional protocol; IQR, interquartile range; OP, optimized protocol

a Indicates statistical significance.

## Discussion

The present study aimed to enhance the preservation of marginal porcine slaughterhouse hearts using a further optimized, cardioprotective perfusion approach compared to a conventional protocol. Our findings demonstrate that myocardial performance was significantly improved, while functional decline was attenuated during 4 hours of normothermic ESHP, indicating improved preservation. Notably, these results were obtained using very marginal hearts from slaughterhouse pigs that had suffered a warm ischemic and an additional cold ischemic insult, suggesting that our findings might be enhanced in hearts of optimal quality. Enhancing preservation not only holds promise for improving clinical outcomes after transplantation but also expands the scope of this technique to include biological modification, improved donor-recipient matching, and enable ESHP for logistical purposes (i.e., “park and preserve”), analogous to what is already implemented clinically for lungs, kidneys and livers.^19^, ^20^, ^21^, ^22^

Our optimized perfusion approach can be divided into 3 main areas, being (1) the optimalization of the cardioplegic solution; (2) ameliorating ischemia-reperfusion injury during initial reperfusion; and (3) optimizing the perfusate composition over the course of ESHP.

### Cardioplegia

The initial step toward optimizing perfusion is the administration of a suitable cardioplegic solution that offers adequate protection against the effects of (warm) ischemia. This limits irreversible damage to the myocardium during subsequent static preservation. Ischemia depletes myocardial energy stores, resulting in a range of biochemical and metabolic changes, including intracellular acidosis caused by anaerobic glycolysis.^23^, ^24^, ^25^ This acidic environment activates the Na-H-ion exchanger and results in intracellular sodium accumulation, which cannot be resolved due to the ceased function of the Na-K-ATPase. As a result, the Na-Ca-ion exchanger is activated and intracellular calcium overload ensues. After reperfusion, a large hydrogen ion gradient across the cell membrane exacerbates sodium and calcium influx, causing intracellular calcium overload, cell death, hypercontracture, disruption of mitochondrial integrity, and the formation of reactive oxygen species (ROS).

To prevent this, the composition of the extracellular environment can be altered during ischemia. One approach is to induce hyperkalemia in the extracellular environment using a hyperkalemic cardioplegic solution (i.e., St. Thomass II), which blocks the fast sodium channels associated with the rapid upstroke of the myocardial action potential and induces a depolarized arrest. However, at the membrane potential induced by the potassium concentration (16 mmol/liter), some cellular sodium currents are still maintained, contributing to the adverse cascade of events described above.^26^

By inducing hyperpolarization through the sodium-channel-blocking properties of lidocaine and maintaining hyperpolarization by the potassium-channel-blocking properties of adenosine, passive sodium overload can be prevented.^25^, ^26^ By rendering the solution hypocalcemic, the risk of intracellular hypercalcemia is further reduced.^3^ Furthermore, adenosine and insulin are associated with the activation of certain cardioprotective antiapoptotic pathways that contribute to improved graft preservation, especially in the context of an additional warm-ischemic insult.^25^, ^27^, ^28^

### Mild hypothermia and controlled rewarming

Reperfusion at mild hypothermia followed by controlled rewarming has been shown to have several beneficial effects, including increased mitochondrial respiration and reduced cellular oxidative stress following ischemia.^29^, ^30^ This is also observed in kidneys and livers following a period of static cold preservation.^31^, ^32^, ^33^ The main hypothesis explaining this phenomenon is that mild hypothermic reperfusion allows for the controlled reintroduction of oxygen and nutrients, promoting metabolic recovery, and restoration of ion homeostasis prior to full functional activation of the myofibrillar contractile unit. In addition, the slowed metabolism at lower temperatures results in reduced generation of ROS associated with swift reactivation of mitochondrial oxidative phosphorylation, resulting in decreased reoxygenation injury.^33^ Taken together, these processes contribute to reduced ischemia-reperfusion injury and improved organ preservation.

### Methylprednisolone

ESHP is associated with activation of the innate immune system and development of oxidative stress, which is augmented by ischemia-reperfusion injury and contact of blood cells with nonendothelialized surfaces of the circuit. This results in release of a multitude of cytokines (i.e., IL-1b, IL-6, IL-8, Il-10, TNF-alpha, etc.) and is a key contributor to functional decline during ESHP, presumably due to exacerbated oxidative stress, endothelial dysfunction, and inflammasome formation, resulting in increased myocardial tissue stress.^6^, ^7^ Therefore, interventions to limit inflammation may be beneficial.^4^, ^6^, ^7^ One pragmatic approach is to add an immunosuppressant to the perfusate during ESHP, such as methylprednisolone. This was assessed by Sandha et al,^13^ who reported significantly reduced levels of IL6, IL8, IL1-beta, and IL10, as well as a reduction in myocardial edema formation after 6 hours of ESHP when methylprednisolone was added. Although global functional improvement was not observed, the reduction in edema formation suggests a protective effect on local endothelial cell integrity, which is consistent with other studies.^34^ This finding may have additional relevance to clinical heart transplantation, given the association between endothelial damage and the development of cardiac allograft vasculopathy.^35^, ^36^

### Hemofiltration

Hemofiltration is a method used to manage fluid balance and remove micromolecular components by creating a hydrostatic pressure across a semipermeable membrane. It is often used in critically-ill patients who require renal-replacement therapy. Since the heart is highly metabolically active in a nonphysiologic environment during normothermic ESHP, accumulation of toxic metabolic waste products can be expected without means for excretion. This poses an incremental risk to organ quality, especially during prolonged ESHP. By adding hemofiltration to ESHP, we have introduced a method for perfusate clearance to prevent the accumulation of waste products and thereby stabilize the perfusate composition. Evidence for the benefits of added hemofiltration or similar techniques (e.g., dialysis) comes from our prior positive experience with hemofiltration that resulted in improved myocardial function during ESHP,^37^ as well as reports from other groups investigating hemofiltration during ESHP,^12^ and studies reporting on hemofiltration during ex situ perfusion of livers,^38^, ^39^, ^40^ which resulted in improved perfusate composition and organ viability. Interestingly, studies that assessed the effects of plasma-cross circulation or plasma infusion on organ viability showed similar effects, indicating that continuous purification of the perfusion solution may be a necessary step toward extending ESHP preservation times and improving quality.^11^, ^41^, ^42^

### Pyruvate

Prior research suggests that glucose and fatty acid metabolism may be disrupted during ESHP, resulting in decreased adenosine triphosphate (ATP) production and induction of an energy-depleted state that contributes to functional decline.^4^, ^5^, ^43^ It is therefore hypothesized that optimizing metabolic substrate utilization during ESHP can significantly improve the quality of preservation. Evidence supporting this hypothesis is supplied by Hatami et al, who demonstrated a dramatic increase in myocardial function following a single bolus of the lower level metabolic intermediate pyruvate (5 mmol/liter) after 11 hours of normothermic ESHP.^5^, ^14^ The beneficial effects of pyruvate on cardiac preservation can be attributed to its ability to enter cells passively and be incorporated in the tricarboxylic acid cycle as acetyl-CoA, thereby facilitating ATP production. Pyruvate has also been shown to reduce ROS and can be used as an anaplerotic substrate to replenish tricarboxylic acid cycle intermediates, which may become depleted during extended ESHP when glucose is the only metabolic substrate.^44^, ^45^, ^46^, ^47^, ^48^, ^49^ These properties may contribute to the improved myocardial function observed when pyruvate is supplied in addition to glucose and insulin during ESHP.

### Limitations

Limitations to the current study include the limited perfusion time of 4 hours, the absence of a transplantation model, and the fact that we did not conduct more extensive analyses of tissue damage patterns between both protocols at histological, immunohistochemical and ultrastructural level. Furthermore, we did not conduct analysis aimed at assessing altered gene expression patterns through transcriptomics or proteomics. We also did not assess the influence of each individual component of the OP on graft preservation. Furthermore, although the slaughterhouse model is attractive from an ethical and financial point of view, the translational value might be questionable since it does not entirely simulate common clinical organ donation scenarios. However, we were able to design a protocol for optimal preservation of these marginal hearts by combining separate concepts from individual studies and test it as a “proof-of-concept” to establish a foundation for further research into extending and optimizing preservation.

### Future perspectives

The introduction of ESHP has revolutionized the field of heart transplantation; however, there is still substantial room for improvement to fully realize its potential. ESHP offers a unique opportunity for selective organ modulation without systemic side-effects, which extends beyond mere organ preservation. Regardless of the intended use of ESHP, it is essential to continue research into the physiological principles of isolated organ perfusion and improve preservation methods to mitigate the observed functional decline in currently employed ESHP protocols. These studies should focus on breaking the downward spiral of energy deficiency, endothelial activation, and edema formation that result in graft failure. Understanding the pathophysiological mechanisms behind these phenomena would enable selective modulation of these processes and help to create a normo-physiological environment for heart storage that enables prolonged preservation and extend the applicability of ESHP beyond organ preservation.

In conclusion, we designed a more optimized, cardioprotective protocol for ESHP resulting in superior myocardial function and limited decline during 4 hours of normothermic perfusion. Future research should focus on extending preservation times using this protocol and improve our understanding of the pathophysiological mechanisms during ESHP.

## Disclosure statement

Sjoerd van Tuijl is an employee of LifeTec Group B.V. The other authors have no conflict of interest to report.

This paper is supported by the partners of Regenerative Medicine Crossing Borders (RegMed XB), a public-private partnership that uses regenerative medicine strategies to cure common chronic diseases.

This collaboration project is financed by the Dutch Ministry of Economic Affairs by means of the public-private partnership allowance made available by the Top Sector Life Sciences & Health to stimulate public-private partnerships.

## Author contributions

M.V. and S.T. designed the protocol and conducted the experiments. M.V. and E.B. drafted the manuscript. S.T., S.K.g.D., S.D.J., J.S., P.D., and N.v.d.K. supervised the experiments and provided feedback on prior versions of the manuscript. All authors approved of the final manuscript before submission.
